# Explainable machine learning approach to predict extubation in critically ill ventilated patients: a retrospective study in central Taiwan

**DOI:** 10.1186/s12871-022-01888-y

**Published:** 2022-11-14

**Authors:** Kai-Chih Pai, Shao-An Su, Ming-Cheng Chan, Chieh-Liang Wu, Wen-Cheng Chao

**Affiliations:** 1 College of Engineering, Tunghai University, Taichung, Taiwan; 2 Artificial Intelligence Center, Tunghai University, Taichung, Taiwan; 3 Division of Critical Care and Respiratory Therapy, Department of Internal Medicine, Taichung Veterans General Hospital, Taichung, Taiwan; 4 Central Taiwan University of Science and Technology, Taichung, Taiwan; 5 Department of post-Baccalaureate Medicine, College of Medicine, National Chung Hsing University, Taichung, Taiwan; 6 College of Science, Tunghai University, Taichung, Taiwan; 7 Department of Critical Care Medicine, Taichung Veterans General Hospital, Taichung, Taiwan; 8 Department of Automatic Control Engineering, Feng Chia University, Taichung, Taiwan; 9 Big Data Center, Chung Hsing University, Taichung, Taiwan

**Keywords:** Mechanical ventilation, Extubation, Machine learning, Explanation, Critically ill patients

## Abstract

**Background:**

Weaning from mechanical ventilation (MV) is an essential issue in critically ill patients, and we used an explainable machine learning (ML) approach to establish an extubation prediction model.

**Methods:**

We enrolled patients who were admitted to intensive care units during 2015–2019 at Taichung Veterans General Hospital, a referral hospital in central Taiwan. We used five ML models, including extreme gradient boosting (XGBoost), categorical boosting (CatBoost), light gradient boosting machine (LightGBM), random forest (RF) and logistic regression (LR), to establish the extubation prediction model, and the feature window as well as prediction window was 48 h and 24 h, respectively. We further employed feature importance, Shapley additive explanations (SHAP) plot, partial dependence plot (PDP) and local interpretable model-agnostic explanations (LIME) for interpretation of the model at the domain, feature, and individual levels.

**Results:**

We enrolled 5,940 patients and found the accuracy was comparable among XGBoost, LightGBM, CatBoost and RF, with the area under the receiver operating characteristic curve using XGBoost to predict extubation was 0.921. The calibration and decision curve analysis showed well applicability of models. We also used the SHAP summary plot and PDP plot to demonstrate discriminative points of six key features in predicting extubation. Moreover, we employed LIME and SHAP force plots to show predicted probabilities of extubation and the rationale of the prediction at the individual level.

**Conclusions:**

We developed an extubation prediction model with high accuracy and visualised explanations aligned with clinical workflow, and the model may serve as an autonomous screen tool for timely weaning.

**Supplementary Information:**

The online version contains supplementary material available at 10.1186/s12871-022-01888-y.

## Background

Mechanical ventilation (MV) is a life-saving and essential organ support system in intensive care units (ICU), and it is estimated that approximately one million patients required MV in the United States in 2017, with an 83% increase in incidence from 249 to 455 cases per 100,000 person-year in the past two decades [1, 2]. Accumulating studies have shown that delayed weaning from MV has deleterious impacts on critically ill ventilated patients [3, 4]. Notably, weaning, consisting of breathing trial and extubation, requires teamwork among the critical care staff interpretation of multi-disciplinary data in the weaning process [5–7]. Recently, a number of studies have employed artificial intelligence (AI), mainly machine learning (ML), to predict the initiation of breathing trial as well as extubation failure/success, but the study focuses on predicting the time of extubation is still lacking [8–12]. We hence aim to use an explainable ML approach and a real-world critical care dataset for the development of an extubation prediction model.

Explanation of AI models is increasingly recognised as a substantial component with regard to the landing of AI models [13, 14]. Our recent studies have shown that explainable ML can be used to predict the 30-day mortality among critically ill influenza patients, long-term mortality in critically ill ventilated patients, and weaning outcome in patients requiring prolonged mechanical ventilation at Taichung Veterans General Hospital (TCVGH), a tertiary referral centre in central Taiwan [15–17]. In the present study, we aim to establish an extubation prediction model in accordance with the workflow in critical care through using an explainable ML approach and the critical care database at TCVGH.

## Methods

### Ethical approval

The study was performed in accordance with the Declaration of Helsinki. The Institutional Review Board of Taichung Veterans General Hospital approved this study (TCVGH: CE20249B and SE22143B). We used the anonymised electronic medical record (EMR) at TCVGH, and informed consent was waived by the Institutional Review Board of Taichung Veterans General Hospital.

### Critical care database at TCVGH

The critical care database in this study was established through using data from the data warehouse at TCVGH, a Taiwanese referral centre with approximately 1,500 beds and six ICUs in central Taiwan. Subjects who were admitted to ICUs between 2015 and 2019 were enrolled for analyses, and data of the first ICU admission was used among those with ICU admission more than one time. We categorised the data into main clinical domains in accordance with the clinical workflow in critical care, and the four main clinical domains consisted of consciousness/awareness domain, fluid balance domain, ventilatory function domain, and physiological parameter domain. In detail, the consciousness domain contained the Glasgow coma scale (GCS) as well as the Richmond Agitation Sedation Scale (RASS) which is an essential scale to measure the agitation or sedation level in critically ill patients, fluid balance domain included administered fluid, urine output as well as feeding amount, ventilatory parameter domain consisted of peak airway pressure (Ppeak), mean airway pressure (MAP), ventilator-day as well as respiratory rate, and physiology domain which was composed of heart rate [18].

### Machine learning models

We employed five machine learning (ML) models, including extreme gradient boosting (XGBoost), categorical boosting (CatBoost), light gradient boosting machine (LightGBM), random forest (RF) and logistic regression (LR), and the ratio between training/testing was 80/20 in this study (see supplemental Fig. 1 for the flow diagram of the study). Given that we aimed to predict weaning one day prior to extubation by using the two-day data (data of two and three days prior to extubation), the feature window and prediction window were hence 48 h and 24 h, respectively (Supplemental Fig. 2 for details regarding the data time frame in this study).

**Fig. 1:**
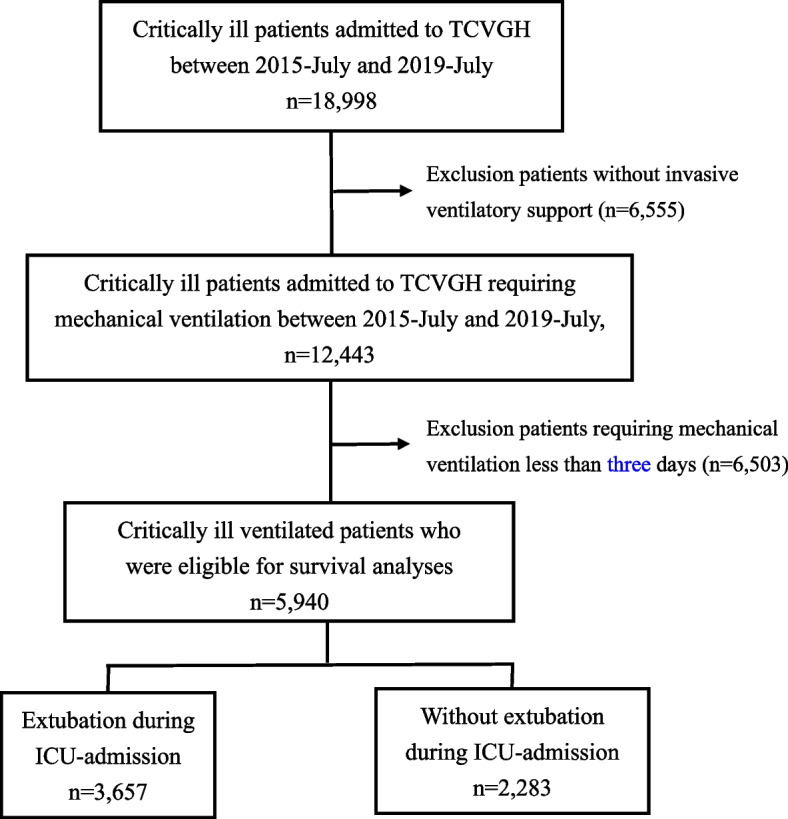
Flowchart of subject enrollment. Abbreviations: TCVGH, Taichung Veterans General Hospital; ICU, intensive care unit

**Fig. 2:**
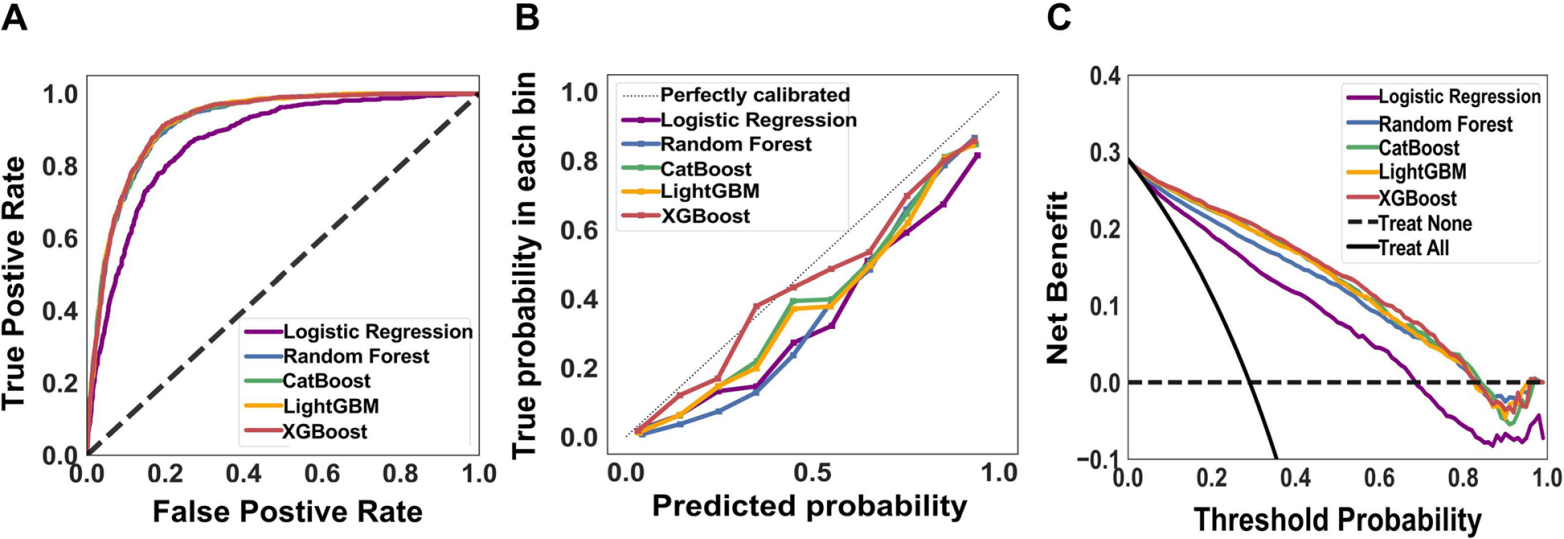
The performance of distinct machine learning models to predict extubation. Receiver operating characteristic curves (**A**), Calibration curves (**B**), Decision curve analyses (**C**). Area under curve (XGBoost 0.921, LightGBM 0.921, CatBoost 0.920, Random Forest 0.918, Logistic Regression 0.868)

With regards to data preprocessing, the physicians set the plausible range of each variable, and the missing data were imputed by the average value of each variable (Supplemental Table 1 for the plausible range and proportion of missing data of the top 20 variables with high feature importance). Given that ML models cannot take the time factor into consideration, we inputted the data within the two-day feature window not only individual data of the two days but also the difference between the two days. All of the data were normalised into -1– + 1 prior to analyses. We further applied recursive feature elimination for succinct features and used 20 features to establish the extubation prediction model (Supplemental Fig. 3 for the results of recursive feature elimination analysis). To avoid the potential bias in sampling, we used two sets of data, including the data one day prior to extubation and another random set of data, in patients with extubation and randomly selected five sets of data in patients without extubation. The ratio of datasets labelled with extubation and non-extubation was 1:3.4; therefore, the imbalance issue should be at least partly mitigated. With respect to the explanation, we used a number of visualised tools for explanation at domain-, feature- and individual levels to reduce the potential concern regarding the black-box of ML models. In detail, we quantified the score of feature importance and illustrated the cumulative feature importance in accordance with the main clinical domains. We further used SHAP and PDP plots to show the direction and trend of impacts on the extubation prediction at feature level [19]. In detail, the SHAP summary plot illustrated both the direction and strength of associations between key features and extubation probability and the partial dependence plot (PDP) further showed the marginal effect of the selected key features on the extubation prediction. For the individual-level explanation, we showed extubation probability and used LIME and SHAP force plots for visualising the impact of key features on extubation [20]. In detail, LIME provides an explanation of the proposed classifier through approximating the selected number of key features through applying a locally linear model, and the LIME plot reflects the contribution of key features to the extubation of the selected patient.

**Table 1:** Characteristics of the 5,940 critically ill ventilated patients with and without extubation during ICU-admission

	**All**	**Extubation (-)**	**Extubation ( +)**	***p*-value**
	***N* = 5,940**	***N* = 2,283**	***N* = 3,657**	
**Demographic data**				
Age (years)	66.2 ± 16.2	65.8 ± 16.0	66.4 ± 16.3	0.12
Sex (male)	3799 (64.0%)	1482 (64.9%)	2317 (63.4%)	0.24
Body mass index	24.0 ± 5.0	23.5 ± 4.8	24.0 ± 5.2	< 0.01
**CCI**	2.1 (1.4)	2.1 (1.4)	2.2 (1.5)	0.05
**ICU types**				< 0.01
Medical ICU	2831 (47.7%)	1043 (45.7%)	1788 (48.9%)	
Surgical ICU	1272 (21.4%)	512 (22.4%)	760 (20.8%)	
Neurological ICU	1176 (19.8%)	575 (25.2%)	601 (16.4%)	
Cardiac ICU	399 (6.7%)	110 (4.8%)	289 (7.9%)	
Cardiosurgical ICU	262 (4.4%)	43 (1.9%)	219 (6.0%)	
**Severity score**				
APACHE II	25.7 ± 6.6	26.7 ± 6.8	25.0 ± 6.3	< 0.01
SOFA score	8.5 ± 3.6	9.0 ± 3.9	8.2 ± 3.4	< 0.01
**Outcome**				
ICU-stay (day)	14.7 ± 10.6	17.2 ± 12.0	13.1 ± 9.3	< 0.01
Ventilator-day	12.2 ± 10.8	16.0 ± 12.0	9.7 ± 9.1	< 0.01
Hospital-stay (day)	29.4 ± 16.7	30.3 ± 18.6	28.8 ± 15.4	< 0.01

Data were presented as mean ± standard deviation and number (percentage)

Abbreviations: *CCI* Charlson comorbidity index, *ICU* intensive care unit, *APACHE II* acute physiology and chronic health evaluation II, *SOFA* sequential organ failure assessment

**Fig. 3:**
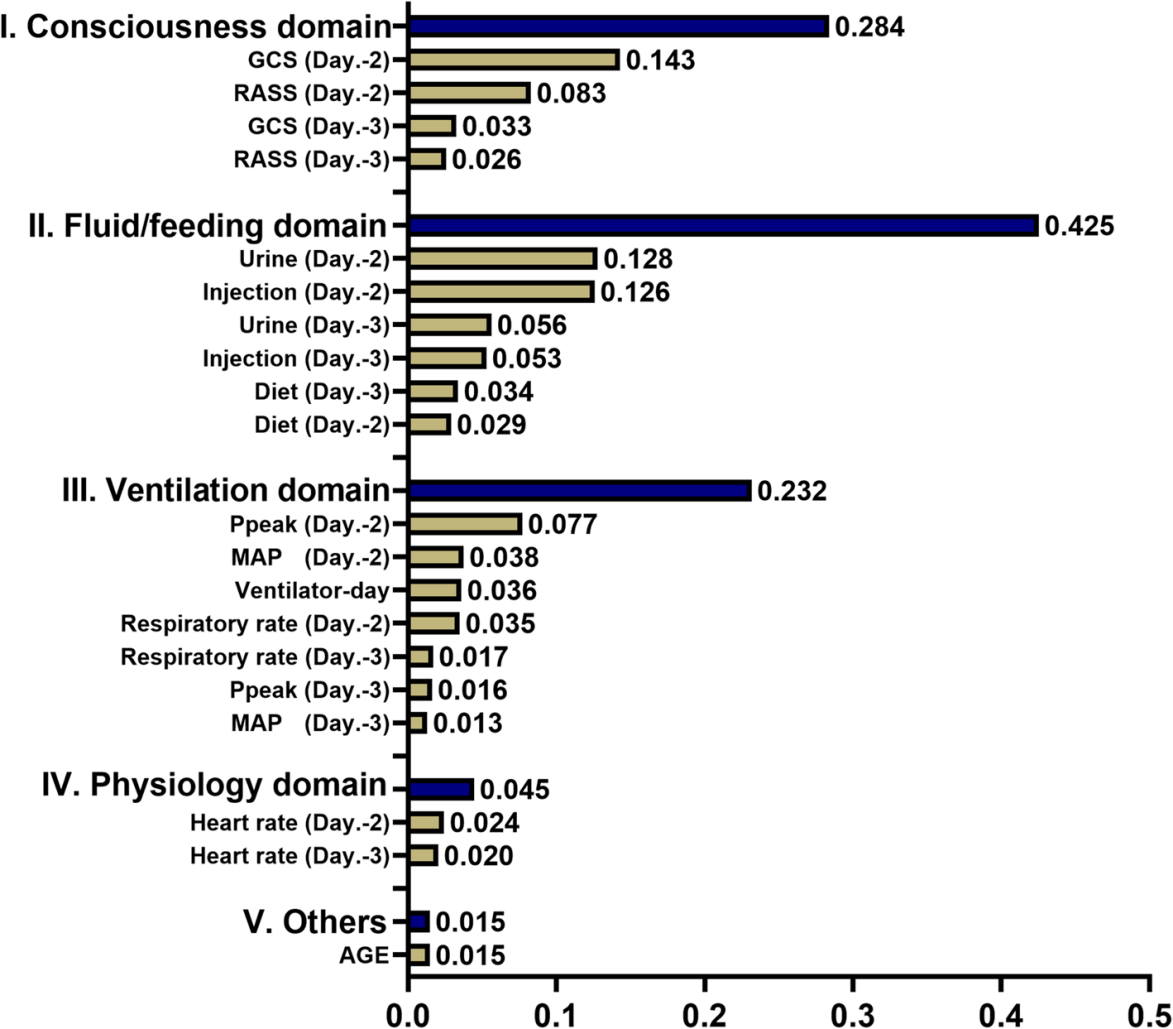
Cumulative relative feature importance of features categorised by working domains in critical care

### Statistical analysis

We presented the continuous data as means ± standard deviations, and categorical data were expressed as frequencies (percentages). Fisher’s exact test and Student’s t-test were used to measure the difference between the two groups. We determined the discrimination, accuracy and applicability of the models in the testing sets by the receiver operating characteristic (ROC) curve analysis, calibration curve as well as decision curve analysis [21, 22]. Python version 3.6 was applied in the present study.

## Results

### Demographic and dynamic data of main domains among enrolled subjects

We enrolled 5,940 critically ill patients requiring mechanical ventilation for more than 48 h, and 65 features were used in the present study (Fig. 1). The mean age of enrolled subjects was 66.2 ± 16.2 years, and 64.0% of them were male. The majority of patients were admitted to the medical ICU, followed by surgical ICU and neurological ICU. Given we excluded those requiring mechanical ventilation for less than 72 h, the enrolled subjects had an apparently high disease severity, with acute physiology and chronic health evaluation (APACHE) II and sequential organ failure assessment (SOFA) scores were 25.7 ± 6.6 and 8.5 ± 3.6, respectively. We found that 61.5% (3657/5940) were extubated during the ICU admission (Supplemental Fig. 4 for the distribution of hospital length of stay and ventilator day). Patients with and without extubation had similar distributions in age, sex, and Charlson comorbidity index. However, those without extubation had a higher APACHE II score (26.7 ± 6.8 vs 25.0 ± 6.3, *p* < 0.01) and SOFA score (9.0 ± 3.9 vs 8.2 ± 3.4) than those with extubation (Table 1). Table 2 shows the dynamic parameters of enrolled patients, and we found that patients with extubation during ICU admission had a continuous improvement of consciousness and decreased sedation status, a gradual decrease in heart rate and administered fluid, and a steady increase in urine output and feeding amount (Table 2).

**Table 2:** Dynamic parameters of critically ill ventilated subjects without and with extubation

	**All**	**Extubation (-)**	**Extubation ( +)**	***p*-value**
	***N* = 5,940**	***N* = 2,283**	***N* = 3,657**	
**Day 1**				
Glasgow coma scale	7.5 ± 4.4	6.8 ± 4.2	8.0 ± 4.4	< 0.001
RASS level	-2.9 ± 1.9	-3.2 ± 1.8	-2.7 ± 1.9	< 0.001
Respiratory rate, per minute	19.2 ± 3.7	19.5 ± 3.8	19.1 ± 3.6	< 0.001
Ppeak, cmH2O	22.3 ± 6.0	22.8 ± 6.2	22.0 ± 6.0	< 0.001
MAP, cmH2O	12.4 ± 3.1	12.6 ± 3.3	12.2 ± 2.9	< 0.001
Heart rate, per minute	93.6 ± 19.2	95.9 ± 20.0	92.2 ± 18.5	< 0.001
Urine output, ml	1248.4 ± 1172.1	1270.1 ± 1243.4	1235.0 ± 1125.4	0.290
Injected fluid, ml	2515.0 ± 1929.8	2634.5 ± 2036.9	2439.7 ± 1855.6	< 0.001
Feeding amount, ml	484.1 ± 437.1	504.2 ± 454.9	471.4 ± 425.1	0.045
**Day 3**				
Glasgow coma scale	9.1 ± 4.7	7.3 ± 4.6	10.2 ± 4.5	< 0.001
RASS level	-2.1 ± 2.0	-2.9 ± 1.9	-1.6 ± 1.8	< 0.001
Respiratory rate, per minute	18.0 ± 4.0	18.8 ± 4.5	17.4 ± 3.6	< 0.001
Ppeak, cmH2O	23.6 ± 5.3	24.7 ± 5.6	22.9 ± 4.9	< 0.001
MAP, cmH2O	12.2 ± 3.4	13.0 ± 3.9	11.8 ± 3.0	< 0.001
Heart rate, per minute	88.4 ± 17.4	91.8 ± 18.8	86.3 ± 16.1	< 0.001
Urine output, ml	2012.8 ± 1354.6	1807.5 ± 1384.7	2139.2 ± 1320.1	< 0.001
Injected fluid, ml	1706.2 ± 1338.8	1929.9 ± 1579.1	1566.8 ± 1142.5	< 0.001
Feeding amount, ml	969.0 ± 508.0	939.5 ± 519.6	986.8 ± 500.2	0.002
**Day 7**				
Glasgow coma scale	10.5 ± 4.5	8.0 ± 4.7	12.0 ± 3.7	< 0.001
RASS level	-2.2 ± 2.4	-3.2 ± 2.2	-1.6 ± 2.3	< 0.001
Respiratory rate, per minute	18.8 ± 3.7	19.0 ± 4.3	18.7 ± 3.4	0.010
Ppeak, cmH2O	23.2 ± 5.6	24.8 ± 5.8	22.0 ± 5.1	< 0.001
MAP, cmH2O	11.9 ± 3.4	12.8 ± 3.8	11.2 ± 2.8	< 0.001
Heart rate, per minute	88.7 ± 16.3	90.5 ± 17.9	87.7 ± 15.2	< 0.001
Urine output, ml	2093.0 ± 1280.6	2005.9 ± 1346.8	2140.5 ± 1240.6	< 0.001
Injected fluid, ml	1168.4 ± 1060.9	1413.7 ± 1231.7	1030.7 ± 923.7	< 0.001
Feeding amount, ml	1160.3 ± 571.4	1122.7 ± 571.5	1180.5 ± 570.4	0.001

Data were presented as mean ± standard deviation. *RASS* Richmond Agitation and Sedation Scale, *Ppeak* peak airway pressure, *MAP* mean airway pressure

### Comparisons among machine learning models

We then compared the performance among the five ML models to predict extubation. In contrast to the relatively low accuracy of LR, we found that XGBoost, LightGBM, CatBoost and RF appeared to have similarly high accuracy, with their AUC were 0.921, 0.921, 0.920 and 0.918, respectively (Fig. 2A). The calibration curve showed good consistency between predicted values and actual observed values, particularly the XGBoost (Fig. 2B). The decision curve analysis further illustrated the well overall net benefits within a relatively wide range of threshold probabilities, particularly in XGBoost and LightGBM (Fig. 2C). We hence used XGBoost in the following analyses.

### Explanation of the model at the domain and feature level

**Fig. 4:**
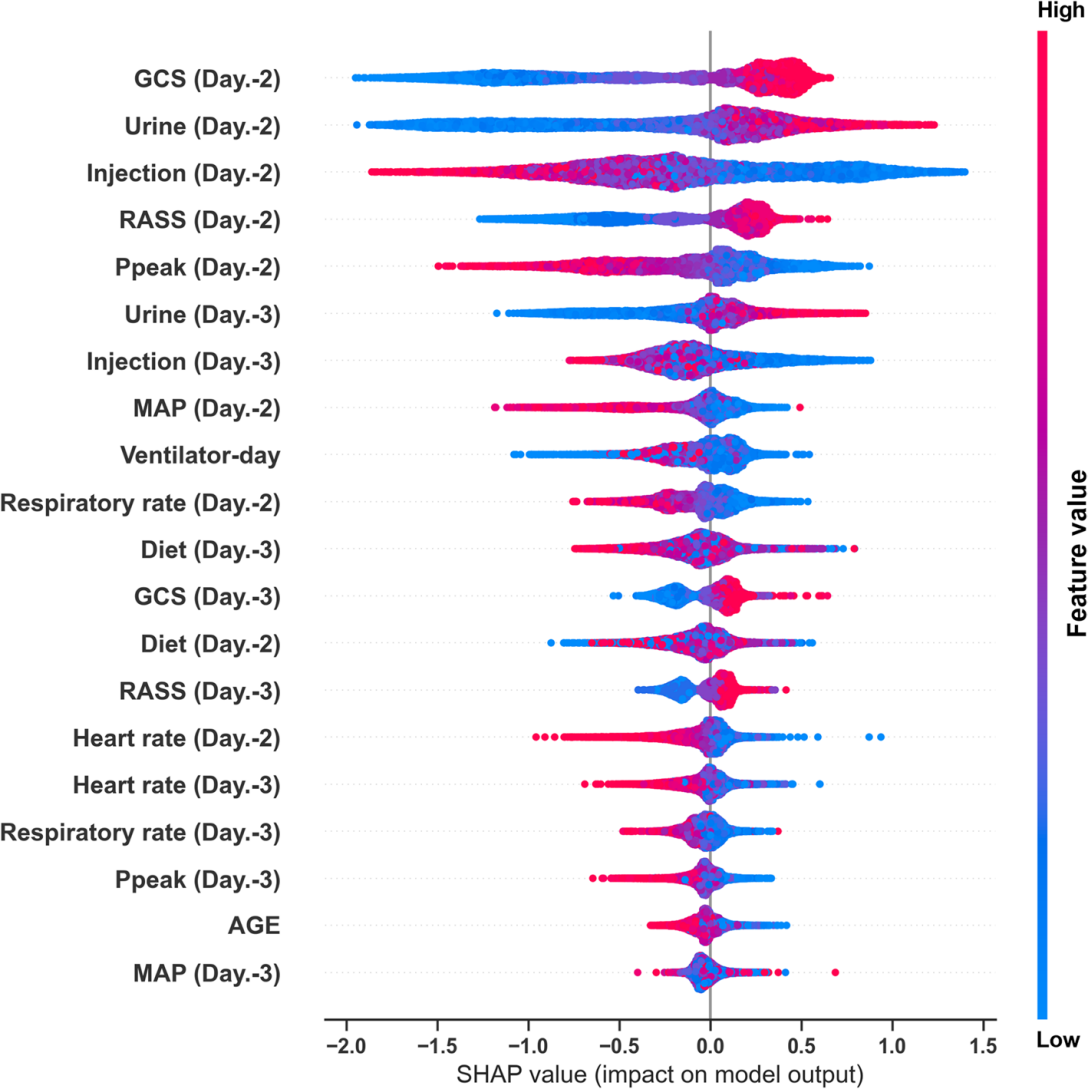
SHAP to illustrate the extubation prediction model at feature level Abbreviation: SHapley Additive exPlanation (SHAP)

**Fig. 5:**
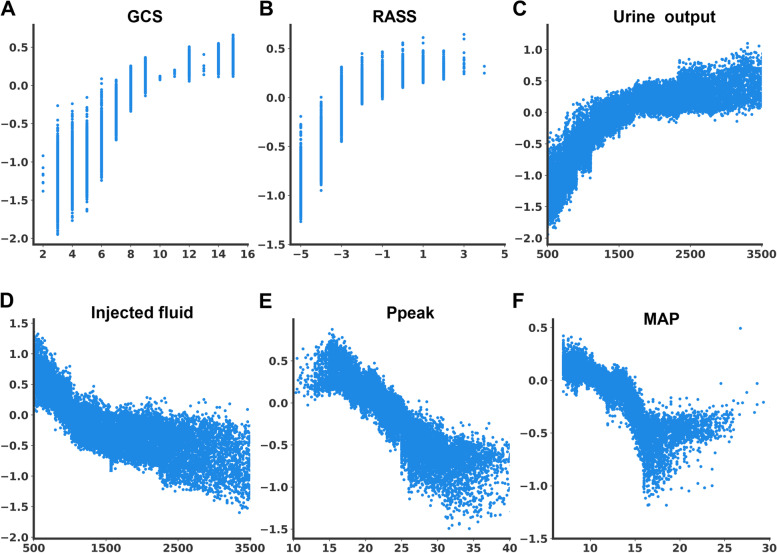
Partial dependence plot by SHAP value in predicting extubation. GCS (A), RASS (B), urine output (C), injected fluid (D), Ppeak (E), MAP (F). Abbreviations: GCS, Glasgow coma scale GCS; RASS, Richmond Agitation and Sedation Scale; Peak, peak airway pressure; MAP, mean airway pressure

We then attempted to illustrate the ML model at the clinical-domain level, feature level, and individual level. We categorised the 20 features by the four clinical domains based on the workflow for management among critically ill ventilated patients (Fig. 3). We found that the cumulative feature importance of the consciousness, fluid balance, ventilatory parameter and physiology domains were 0.284, 0.425, 0.232 and 0.045, respectively (Fig. 3). We then used the SHAP summary plot to demonstrate how these key features affect the probability of extubation (Fig. 4). Using the SHAP summary plot, not only the strength but also the direction of each feature were clearly illustrated. For example, an improved consciousness status, determined by the GCS, as well as increased urine output, was positively associated with a higher probability of extubation one day later, whereas a high requirement for injected fluid was inversely associated with extubation probability. To further elaborate on how each feature affects the probability of extubation within the ML model, we used a PDP plot of the six crucial features, including the consciousness domain (i.e. GCS and RASS), fluid balance domain (i.e. urine output and injected fluid) and ventilatory parameter domain (i.e. Ppeak and MAP) (Fig. 5). Collectively, these visualised interpretations at the domain and feature level based on clinical workflow in critical care should give intuitive explanations of the ML model to the clinician.

### Explanation of the ML model at the individual level

**Fig. 6:**
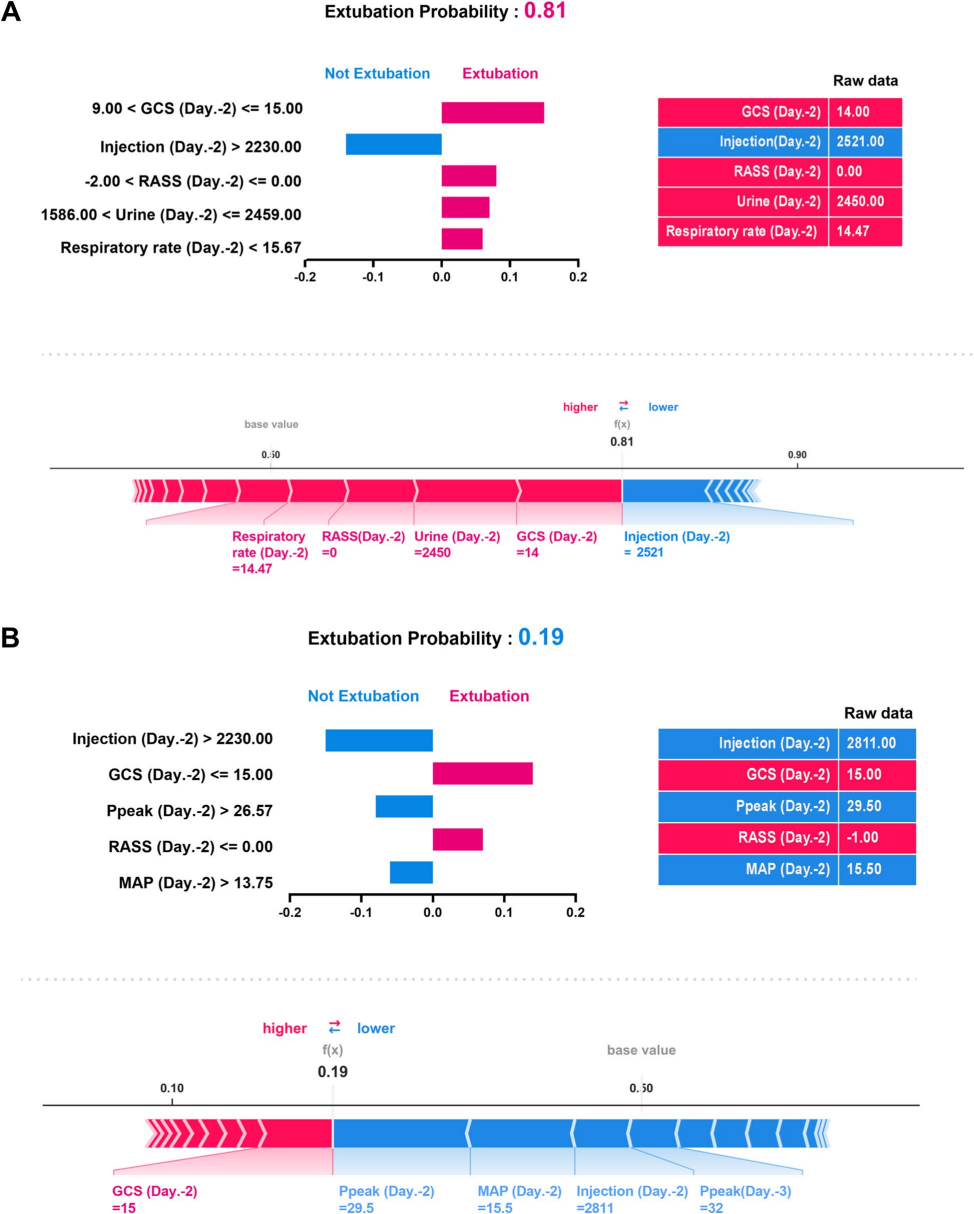
Local interpretable model-agnostic explanations (LIME) and SHAP force plots of two representative individuals. SHapley Additive exPlanation (SHAP)

We then used LIME and SHAP force plots of key features to illustrate the overall impact of key features on the extubation prediction model in two representative individuals. As shown in Fig. 6, the overall predicted probability of extubation, incremental effects on extubation of variables (red), and decremental effects on extubation of variables (blue) of two representative patients were illustrated in the LIME plot (Fig. 6). For example, in case-1, the predicted probability for extubation was relatively high (0.81) due to a number of favourable conditions, consisting of a clear consciousness (GCS: 14 and RASS: 0), high urine output (2450 ml on day -2), and low repiratory rate (14.5 on day -2), although a slightly high injected fluid (2521 ml on day -2). The SHAP force plot illustrated similar findings of aforementioned key features (Fig. 6A). In contrast, the probability of extubation in case-2 was relatively low (0.19) due to a number of unfavourable conditions, including the high injtected fluid (2811 on day -1), high Ppeak (29.50 cmH2O) and MAP (15.5 mg/dL), despite a relatively clear consciousness (GCS: 15 anr RASS -1). SHAP force plot demonstrated similar findings, and the cut-point of each features omitted for the succinct summary (Fig. 6B). Taken together, these explanations at the individual level were in line with the explanation at the feature level and in accordance with the clinical workflow; therefore, the black-box issue should be mitigated through these explanations.

## Discussion

Weaning from mechanical ventilation is an essential but complex issue in critical care and requires the interpretation of multi-domain data in critically ill patients. In the study, we used an explainable ML approach, including domain-based cumulative feature importance, SHAP, PDP and LIME plots, to develop an extubation prediction model with high accuracy and visualised explanations. Notably, the explainability was in line with clinical workflow in critical illness, and we think the proposed extubation prediction model should severe as an autonomous screen tool to aid the clinician for the timely start of breathing trials.

Weaning from mechanical ventilation consists of a patient-tolerated breathing trial followed by extubation, and the start of weaning requires the multi-disciplinary interpretation of data in critical care [3]. Therefore, AI appears to be used for integrating the information in critical care and serves as a decision supporting system to facilliate weaning. Notably, the establishment of the AI model depends on accurate labelling; however, the precise tolerability of distinct breathing trials, mainly T piece and pressure support trial, might somehow be ambiguous and could not be precisely defined in the critical care database [23, 24]. Therefore, we used extubation, which is an explicit, objective and critical medical event in ventilated patients, as the target labelling in the present study to establish an extubation prediction model.

In this study, we found that levels of consciousness/awareness, fluid status relevant features and ventilatory parameters were crucial features with high feature importance to predict extubation one day later, and the finding is in line with the variables of daily screen readiness for spontaneous breathing trial in the respiratory therapist–driven protocol [5]. Indeed, both left- and right-aligned designs can be used to establish the ML models [25]. In brief, left-aligned models predict the incident of the targeted event following a fixed time point, but various time periods among patients may lead to difficulty in the real-world landing of an established model. In contrast, right-aligned models can be used to continuously predict whether the target event will occur after the set time period, so-called real-time or continuous prediction models [25]. Therefore, the right-aligned design in the present study enables the proposed model to serve as an autonomous daily screen system to timely identify patients who were ready for breathing trial and to facilitate the weaning process through early recognition of the potential extubation one day earlier (Supplemental Fig. 3). Furthermore, we think the practical value of the established explainable ML model is high, given that the interpretation of ML models aligns with the real-world workflow in critical care. Recently, the Good Machine Learning Practice for Medical Device Development has incorporated human interpretability into the ML model, the so-called human in the loop [13]. The European Commission also has proposed the ethics guideline for trustworthy AI and includes the need to enhance the explanation of AI-based systems even at the cost of compromised accuracy of the AI-based model [14]. Indeed, safety is a fundamental issue in the field of critical care, and increasing transparency of the model through explanation may at least partly mitigate the concern with respect to the black-box issue [26]. Given that clinicians take accountability with respect to patient safety, the understanding of how the AI systems reach suggested decisions should be crucial in the landing of AI-based systems in the field of critical care [26]. Notably, the design of explanation in accordance with clinical workflow, as we have shown in this study, should further enable clinicians to realise the explainable ML-based model. Nevertheless, it is needed to clarify that to open the black box directly might somehow be difficult, and the current explanation methods are more likely to be post-hoc interpretability of key features through analysing the model after training instead of direct explanations for the entire model [27].

Similar with our study, Chen KH et al. used data of 1,483 patients at three medical ICUs in northern Taiwan and ML approach to establish the shifting of ventilator mode from assisted/controlled mode to spontaneous breath trial, and the accuracy determined by the area under the receiver operating characteristic curve of ML-based model was approximately 0.79 [9]. We think the increased performance of the extubation prediction model in the present study can be attributed not only to a high number of enrolled subjects but also to the explicit target labelling with extubation. Furthermore, the proposed individual-level explanation at distinct time points might serve to continuously monitor the readiness for extubation. In brief, gradual improvement of crucial clinical parameters and steady increase of extubation probability indicates the readiness for extubation of an individual patient (Supplemental Fig. 5). The aforementioned findings further highlight that explanation that is consistent with clinical evidence should enable the clinicians to work with AI, the so-called Human-AI Team [13].

Indeed, feature selection is an essential issue given that a high number of features might be a concern with regard to landing, particularly in the edge device [28, 29]. We hence used recursive feature elimination and found a high accuracy while using the top 20 features in this study (Supplemental Fig. 3) [30]. In line with our findings, Roimi et al. used merely 50 features from 7000 features among the two critical care databases at Beth Israel Deaconess Medical Center and Rambam Health Care Campus to develop an ML-based model to predict bloodstream infections in critically ill patients [31]. Similarly, Jia et al. used 25 features in the Medical Information Mart for Intensive Care (MIMIC) III databases and convolutional neural networks approach to establish a decision support system for suggesting breathing trial, with the accuracy was 0.86 [10]. Moreover, Xie et al. employed merely 9–12 variables to establish an easy-to-use, machine learning-based mortality prediction model through using data of the Medical Information Mart for Intensive Care (MIMIC) III database [32]. These studies and our data demonstrate the potential to establish a model with high accuracy with a reasonable number of features for practical landing.

With respect to the comparison among distinct ML models, we used the Delong test to determine the difference in performance among ML models [33] (Supplemental Table 3). Similar to our previous studies, we found that the tree-based models, including XGBoost, CatBoost, LightGBM and RF, had an apparently better performance compared with those in LR and postulated that the relatively low performance of LR may result from the assumption of linear correlation among features in LR [16, 17]. We also found that XGBoost, LightGBM and CatBoost had a slightly higher performance than that in RF and speculated this minor difference might potentially be attributed to the high flexibility with a number of adjustable hyperparameters of XGBoost, LightGBM and CatBoost. However, we think the difference among XGBoost, Catboost and LightGBM was not the performance but the easy categorical data preprocessing in Catboost as well as the less hardware requirement in LightGBM.

There are limitations in this study. First, this study used a single hospital database, and external validation is warranted to confirm our findings. Second, the retrospect design and the decision of extubation are individualised, but the study hospital is a referral centre in central Taiwan with the administration of intensivists as well as respiratory therapies that might mitigate the concern. Third, the established model predicts the timing of extubation instead of successful weaning (i.e. extubation without re-intubation); however, the proportion of re-intubation in the present study is consistent with previous studies (Supplemental Fig. 6). Fourth, the single imputation method by the average value could potentially lead to a bias in this study.

## Conclusions

Weaning from MV relies on timely recognition of ventilated patients who might be extubated soon and the timely start of the breathing trial. AI is increasingly used in the medical field, but black-box issues remain the main concern, particularly in the field of critical care. We used an explainable ML approach to develop an extubation prediction model with not only high accuracy but also the visualised interpretation of the model in the domain, feature and individual level. The established model may severe as a computer-aided algorithm to detect critically ill ventilated patients who might be extubated one day later and suggest clinicians for a timely start of breathing trial. More prospective studies are required to validate our findings and to land the proposed models in critically ill ventilated patients.

## Supplementary Information

**Additional file 1:** **Supplemental Figure 1.** Flow diagram of the analytic pipeline in the study. **Supplemental Figure 2.** Illustration of the study design and the time frame with right alignment. Subjects were aligned at the alignment point that was extubation-day or one random-day in those without extubation. The data within prediction window (day -3 and day -2 prior to extubation-day) were collected, and the prediction window reflects the time of the prediction ahead of extubation. **Supplemental Figure 3.** Recursive feature elimination to explore the accuracy of model using distinct numbers of the feature to predict extubation in critically ill ventilated patients. **Supplemental Figure 4.** Histograms of hospital length of stay (A) and ventilator-day (B) among enrolled subjects. **Supplemental Figure 5.** Serial explainable predictions of one individual patient. **Supplemental Figure 6.** Extubation outcome of extubation in the 3,657 critically ill ventilated patients with extubation during admission. **Supplemental Table 1.** Plausible range of data and proportion of missing data among the top 20 features with high feature importance. **Supplemental Table 2.** Metrics of performance of distinct machine learning models to predict weaning. **Supplemental Table 3.** Delong test to determine the difference of performance among distinct machine learning models.

## Data Availability

All of the data and materials are provided in the manuscript and the supplemental data. The code has been put in public Github, and is available via https://github.com/GitTerrySu/Predict-extubation.
