# Supplementary material for: Explainable machine learning approach to predict extubation in critically ill ventilated patients: a retrospective study in central Taiwan
Source: BMC Anesthesiol. 2022 Nov 14;22:351. doi: 10.1186/s12871-022-01888-y (PMC9664699; doi:10.1186/s12871-022-01888-y)
Supplement: Supplementary file 1 — Additional file 1: Supplemental Figure 1. Flow diagram of the analytic pipeline in the study. Supplemental Figure 2. Illustration of the study design and the time frame with right alignment. Subjects were aligned at the alignment point that was extubation-day or one random-day in those without extubation. The data within prediction window (day -3 and day -2 prior to extubation-day) were collected, and the prediction window reflects the time of the prediction ahead of extubation. Supplemental Figure 3. Recursive feature elimination to explore the accuracy of model using distinct numbers of the feature to predict extubation in critically ill ventilated patients. Supplemental Figure 4. Histograms of hospital length of stay (A) and ventilator-day (B) among enrolled subjects. Supplemental Figure 5. Serial explainable predictions of one individual patient. Supplemental Figure 6. Extubation outcome of extubation in the 3,657 critically ill ventilated patients with extubation during admission. Supplemental Table 1. Plausible range of data and proportion of missing data among the top 20 features with high feature importance. Supplemental Table 2. Metrics of performance of distinct machine learning models to predict weaning. Supplemental Table 3. Delong test to determine the difference of performance among distinct machine learning models. [file 12871_2022_1888_MOESM1_ESM.pdf]

Supplemental Table 1. Plausible range of data and proportion of missing data among the top 20 features with high feature importance

Variables	Plausible range	Proportion of missing data
**Age, years**	1-100	0.0%
**Ventilator-day**	1-60	0.0%
**Day -2**		
**GCS**	3-15	1.3%
**Urine output, ml**	0-5,000	0.0%
**Injection amount, ml**	0-10,000	0.0%
**Diet amount, ml**	0-3,000	0.0%
**RASS level**	-5–+4	1.3%
**Ppeak, cmH2O**	0-50	3.6%
**Respiratory rate, /min**	0-40	0.2%
**MAP, cmH2O**	10-40	3.9%
**Heart rate, /min**	0-300	0.2%
**Day -3**		
**GCS**	3-15	0.5%
**Urine output, ml**	0-5,000	0.0%
**Injection amount, ml**	0-10,000	0.0%
**Diet amount, ml**	0-3,000	0.0%
**RASS level**	-5–+4	1.2%
**Ppeak, cmH2O**	0-50	2.7%
**Respiratory rate, /min**	0-40	0.0%
**MAP, cmH2O**	10-40	2.8%
**Heart rate, /min**	0-300	0.0%

Abbreviations: GCS, Glasgow coma scale; RASS, Richmond agitation-sedation scale; Ppeak, peak airway pressure; MAP, mean airway pressure.

Supplemental Table 2. Metrics of performance of distinct machine learning models to predict weaning

	Models	Precision	Specificity	Sensitivity	F-1	Brier Score	Accuracy[a]
Validation (80%) (5-fold cross validation)	LR	0.602 ± 0.012	0.777 ± 0.006	0.818± 0.008	0.693 ± 0.010	0.144 ± 0.002	0.793 ± 0.004
	RF	0.660 ± 0.013	0.810 ± 0.010	0.891 ± 0.009	0.757 ± 0.008	0.116 ± 0.004	0.834 ± 0.008
	CatBoost	0.695 ± 0.010	0.846 ± 0.003	0.853 ± 0.002	0.766 ± 0.006	0.106 ± 0.004	0.848 ± 0.002
	LightGBM	0.710 ± 0.010	0.858 ± 0.002	0.842 ± 0.003	0.771 ± 0.006	0.102 ± 0.003	0.854 ± 0.001
	XGBoost	0.732 ± 0.011	0.878 ± 0.006	0.806± 0.008	0.767 ± 0.009	0.101 ± 0.003	0.857± 0.006
Testing (20%)	LR	0.599	0.777	0.815	0.691	0.148	0.788
	RF	0.665	0.818	0.881	0.758	0.118	0.837
	CatBoost	0.688	0.844	0.842	0.757	0.108	0.843
	LightGBM	0.692	0.848	0.839	0.759	0.105	0.845
	XGBoost	0.720	0.873	0.798	0.757	0.103	0.852

[a](TP+TN) / (TP+FN+TN+FP). Abbreviation: LR, logistic regression; RF, random forest, CatBoost, categorical boosting; LightGBM, light gradient boosting machine; XGBoost, Extreme gradient boosting.

Supplemental Table 3. Delong test to determine the difference of performance among distinct machine learning models

	XGBoost	RF	LR	CatBoost	LightGBM
**XGBoost**	NA	<0.01	<0.01	0.25	0.21
**RF**	<0.01	NA	<0.01	0.011	<0.01
**LR**	<0.01	<0.01	NA	<0.001	<0.01
**CatBoost**	0.25	0.01	<0.01	NA	0.06
**LightGBM**	0.21	<0.01	<0.01	0.06	NA

Abbreviation: XGBoost, Extreme gradient boosting; RF, random forest; LR, logistic regression; CatBoost, categorical boosting; LightGBM, light gradient boosting machine; NA, not available.

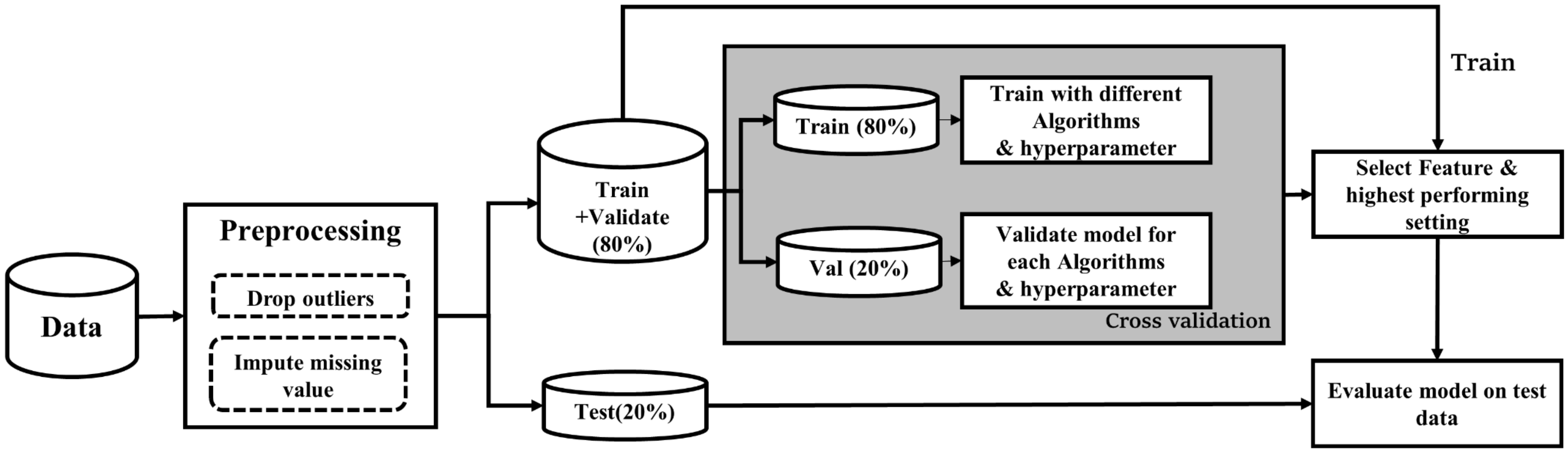

Supplemental Figure 1. Flow diagram of the analytic pipeline in the study

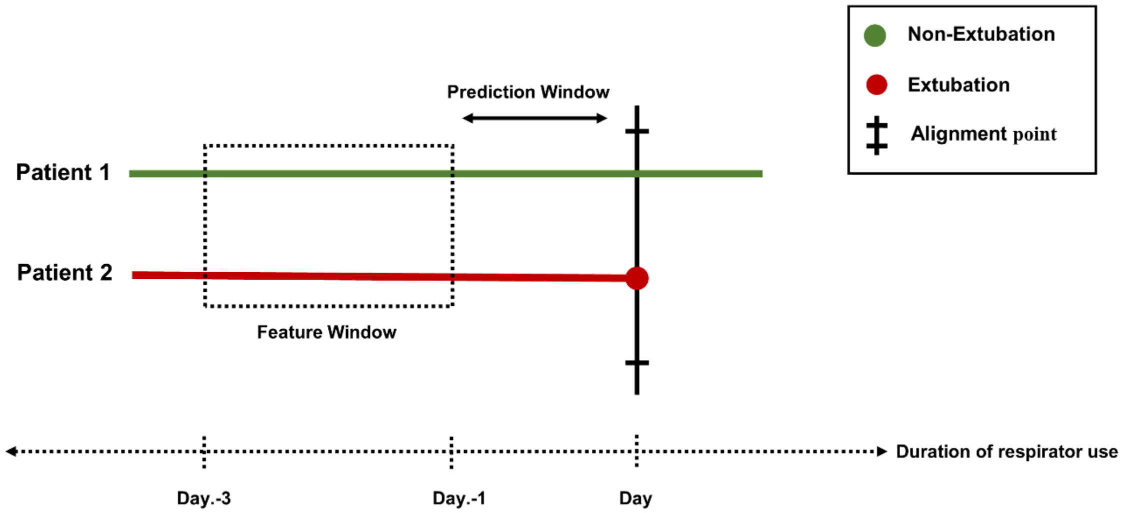

Supplemental Figure 2. Illustration of the study design and the time frame with right alignment. Subjects were aligned at the alignment point that was extubation-day or one random-day in those without extubation. The data within prediction window (day -3 and day -2 prior to extubation-day) were collected, and the prediction window reflects the time of the prediction ahead of extubation.

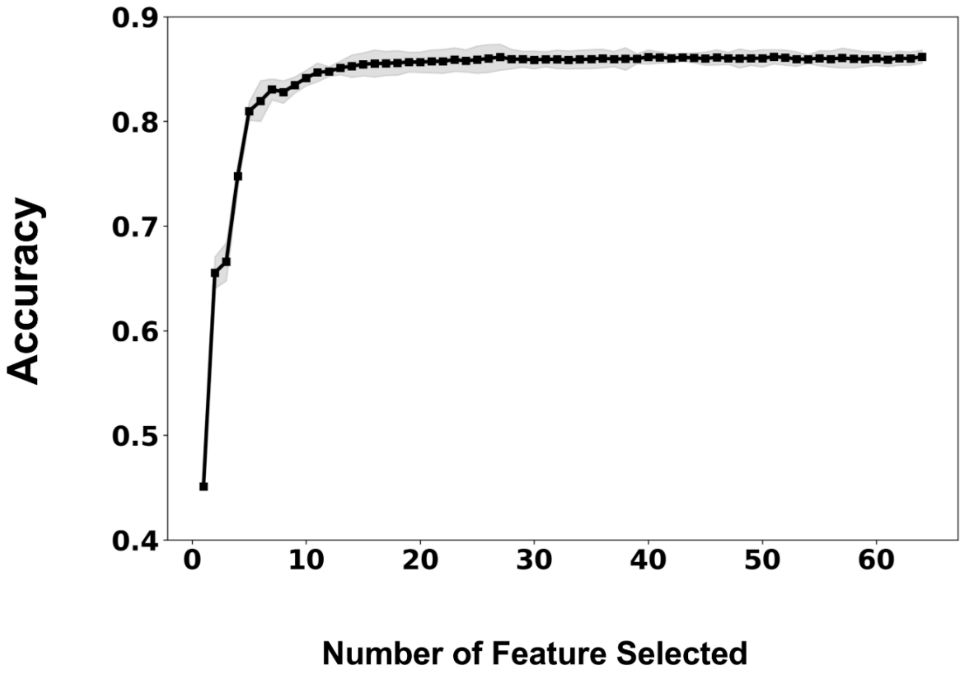

Supplemental Figure 3: Recursive feature elimination to explore the accuracy of model using distinct numbers of the feature to predict extubation in critically ill ventilated patients

A

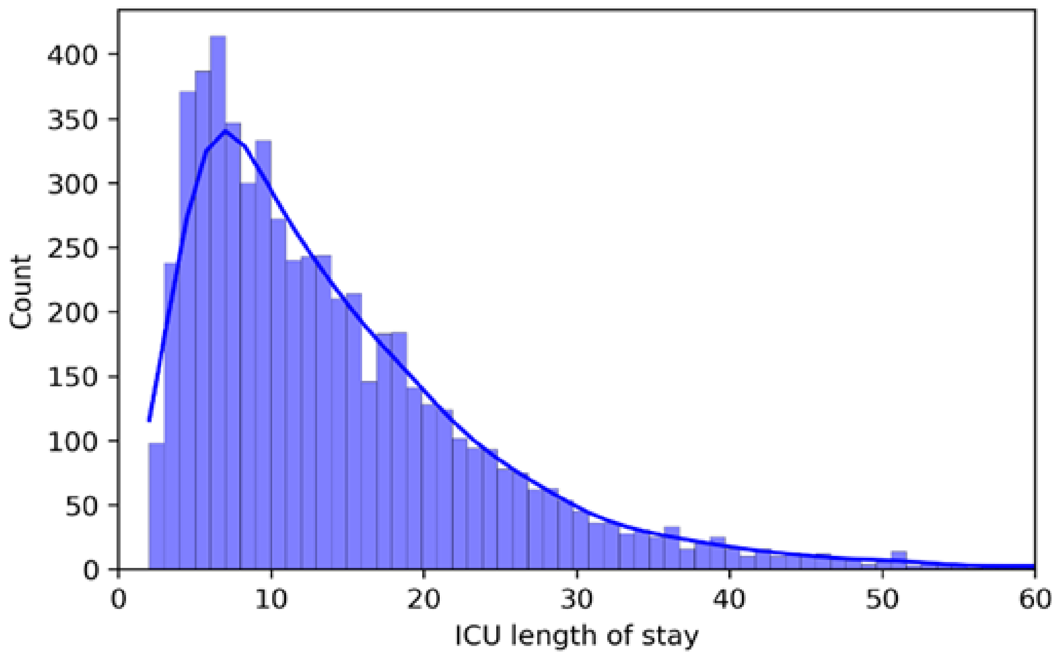

B

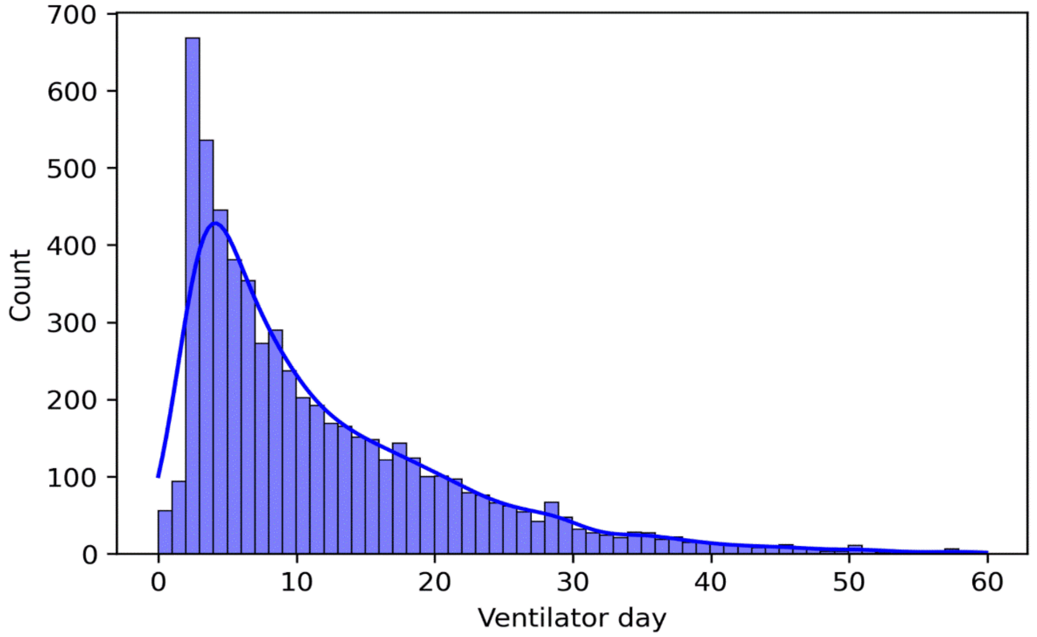

Supplemental Figure 4. Histograms of hospital length of stay (A) and ventilator-day (B) among enrolled subjects.

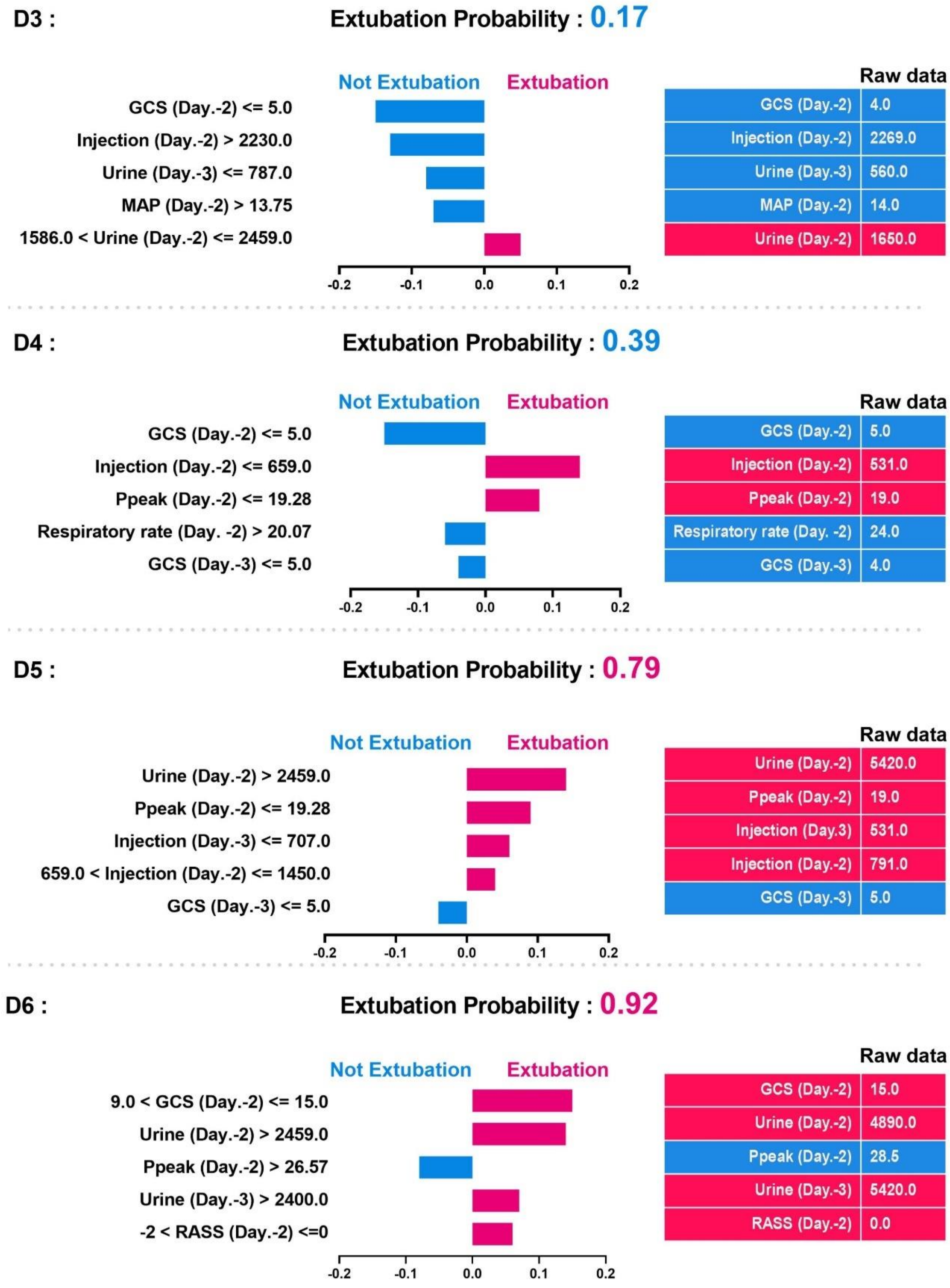

Supplemental Figure 5. Serial explainable predictions of one individual patient

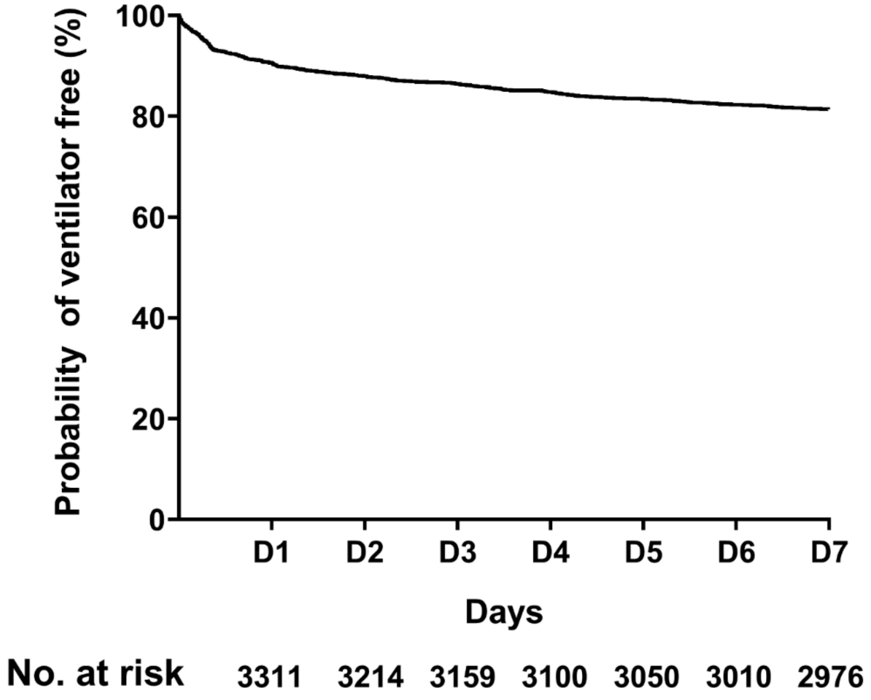

Supplemental Figure 6. Extubation outcome of extubation in the 3,657 critically ill ventilated patients with extubation during admission
